# The Impact of COVID-19 Pandemic on Rehabilitation Services in a Tertiary Care Hospital in the Eastern Region of Saudi Arabia: A Single-Center Study

**DOI:** 10.7759/cureus.18303

**Published:** 2021-09-26

**Authors:** Amara Ilyas, Asim Naiz, Turki Abualait, Shahid Bashir

**Affiliations:** 1 Physical Medicine and Rehabilitation, King Fahad Specialist Hospital, Dammam, SAU; 2 Neuroscience, Neuroscience Center, King Fahad Specialist Hospital, Dammam, SAU; 3 College of Applied Medical Sciences, Imam Abdulrahman Bin Faisal University, Damamm, SAU

**Keywords:** covid-19, pandemic, rehabilitants, speech, physiotherapy, occupational therapy, help-seeking behavior

## Abstract

Objective

In this retrospective study, we aimed to investigate the impact of the coronavirus disease 2019 (COVID-19) pandemic on rehabilitation services including outpatient rehabilitation clinic visits, speech therapy, and occupational therapy and physiotherapy sessions.

Materials and methods

This was a retrospective, observational, single-center study, which included all patients who presented to the rehabilitation clinic at the King Fahad Specialist Hospital, Dammam (KFSHD) from January to August 2020 (study period), and they were matched with parallel groups from 2017 to 2019 (control period).

Results

A total number of 1,435 patient visits were recorded in the outpatient rehabilitation clinics during the study period as compared to the 1,496 patient visits during the control period. However, the number of patients seen by the speech therapists, physiotherapists, and occupational therapists significantly came down from 482, 963, and 171, respectively in the control period to 77, 218, and 130, respectively in the study period. The occupational therapy, physiotherapy, and speech therapy services were significantly affected during the COVID-19 pandemic.

Conclusions

Our findings revealed the negative consequences of the COVID-19 pandemic on the outpatient rehabilitation services, which was reflected in the sharp decline in the number of patients who attended the rehabilitation clinics as well as in the reduced number of sessions. The rehabilitation facilities should be better prepared for such pandemics in the future to deliver appropriate and timely rehabilitation programs with a patient-centered approach.

## Introduction

Rehabilitation is an essential component in healthcare and medical management, and it helps in enhancing functional capacity, promoting health and wellness, preventing disease, and achieving the best possible outcomes overall [1,2]. Rehabilitation is crucial to improve the functional outcome for community reintegration for people with physical limitations (disabilities). The World Health Organization (WHO) defines rehabilitation as “a set of interventions needed when a person is experiencing or is likely to experience limitations in everyday functioning due to aging or a health condition, including chronic diseases or disorders, injuries or traumas” [3]. The ultimate goal of rehabilitation is to enhance an individual’s functional capability, promote independence and wellbeing, and improve the quality of life [1]. Therefore, rehabilitation services should be offered at different levels of medical care such as primary, secondary, and tertiary, covering in-patient and outpatient services and through multiple phases of care: acute, post-acute, and chronic or long-term care [4,5]. A multidisciplinary team from different professions including a physiatrist, physiotherapist, occupational therapist, speech and language pathologist, psychologist, orthotist and prosthetist, rehabilitation nurse, social worker, and dietician should ideally deliver patient-centered multimodal rehabilitation services aiming at a return to normal life for the patient and regaining functional independence.

The world is currently experiencing a major healthcare crisis in the form of the ongoing coronavirus disease 2019 (COVID-19) pandemic, and it has heavily affected global healthcare; similarly, it has affected the rehabilitation of people with functional limitations [6,7]. The WHO report has highlighted that the novel coronavirus has quickly spread across the globe, infecting more than 222 million people and causing the death of around 4.7 million individuals [8]. This pandemic has imposed a huge challenge on healthcare systems around the world. In the Kingdom of Saudi Arabia (KSA), the COVID-19 virus has gradually spread across the country and the confirmed COVID-19 cases have risen to 546,479 [8,9]. There is a major concern that the COVID-19 pandemic might have affected patients’ health-seeking behavior with respect to other conditions as well, particularly chronic diseases. Evidence highlights that when rehabilitation services are cut down or unavailable, risks are encountered at a higher rate in healthcare, and quality of life and health outcomes decline [10,11,12]. Thus, rehabilitation should be considered a crucial service along with other medical services and should be offered throughout the pandemic time [13]. During the COVID-19 pandemic, rehabilitation services have been rolled back or stopped in most hospitals, which has had a significant impact on patients with disabilities, chronic health conditions, acute diseases, or severe trauma; it has even affected COVID-19 patients in the acute phase, often leading to devastating consequences [14]. Hence, in this study, we hypothesized that COVID-19 has affected rehabilitation services (outpatient rehabilitation clinic, speech, physiotherapy, and occupational therapy clinics), and we examined the hypothesis further by analyzing data from a single tertiary care center's computerized database before and during the outbreak of COVID-19.

## Materials and methods

This was a retrospective observational study that included outpatients who attended rehabilitation clinics between 2017-2020 at the King Fahad Specialist Hospital, Dammam (KFSHD).

Data source

Data were retrieved from the central computerized database [MedicaPlus (MedicaCaller.exe)] of the sole tertiary care center at KFSHD in the Eastern Region of KSA. The data were reviewed and reported by rehabilitation consultants (AN and IA) and analyzed by another co-author (SB). The center provides inpatient and outpatient rehabilitation services. However, data were also included from outpatient clinics of rehabilitation physicians, speech therapists, and inpatient services of physiotherapists and occupational therapists. The study period was between January and August 2020, and the data were compared with that of the control period from January to August, from 2017 to 2019. All adult and pediatric patients who attended outpatient rehabilitation clinics were included in the study, while inpatient admissions were excluded.

Data analysis

The SPSS Statistics version 22.0 (IBM, Armonk, NY) was used for data analysis. Continuous variables were assessed for normality and were described using mean and standard deviation, and median with interquartile range (IQR), as appropriate. An independent sample t-test was used to compare the means of normally distributed data.

## Results

**Figure 1:**
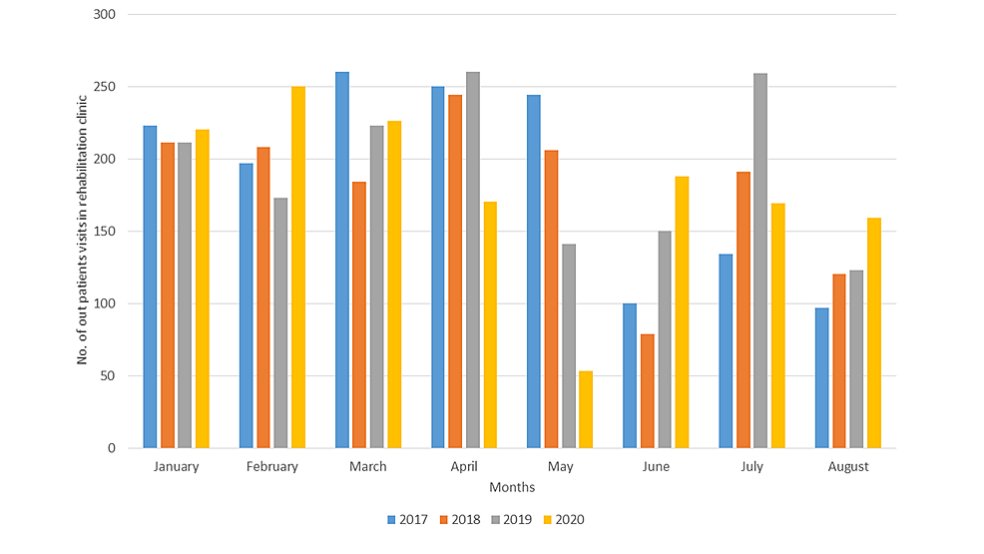
The numbers of patients in the rehabilitation clinic during selected periods in 2020 compared with the same periods in 2017, 2018, and 2019

The number of patients who visited the rehabilitation clinic is shown in Figure 1. The independent t-test showed no significant differences between 2020 and 2017 (2020 median: 210, IQR: 125.5-247; 2017 median: 179, IQR: 166.5-207), between 2020 and 2018 (2018 median: 198.5, IQR: 168-207), or between 2020 and 2019 (2019 median: 192, IQR: 147.7-241). The total number of outpatient visits to rehabilitation clinics decreased during the study period (January-August 2020) compared to the same periods in previous years (2017, 2018, and 2019).

**Figure 2:**
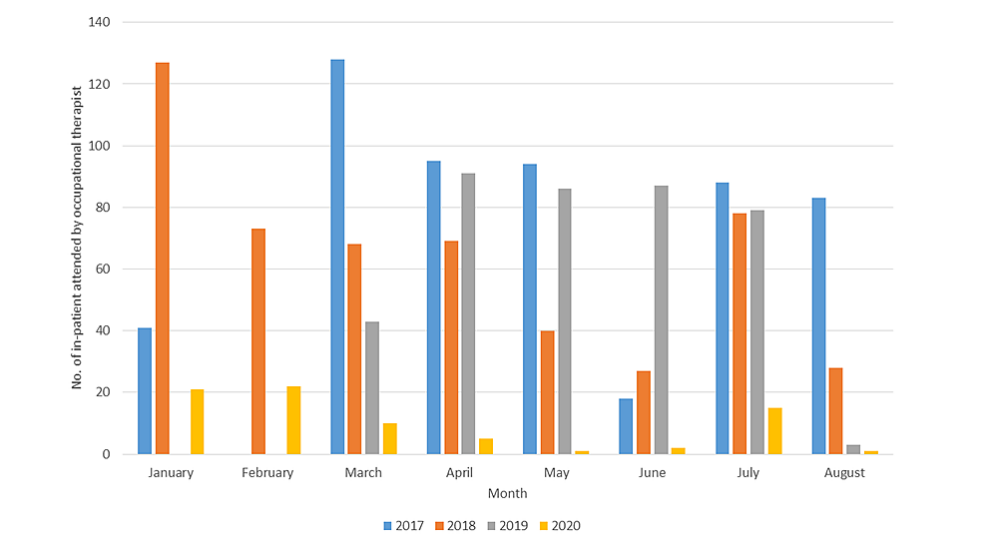
The number of patients receiving occupational therapy during selected periods in 2020 compared with the same periods in 2017, 2018, and 2019

However, a significantly lower proportion of patients received occupational therapy during the study period in 2020 than during the control periods in 2017, 2018, and 2019 (Figure 2). There was a difference in the proportion of patients who were admitted and required occupational therapy in 2017 (median: 88, IQR: 62-94.75, p<0.000), 2018 (median: 68.5, IQR: 37-71, p<0.000), 2019 (median: 61, IQR: 2.25-86.5, p=0.02), and 2020 (median: 7.5, IQR: 1.75-12.5).

**Figure 3:**
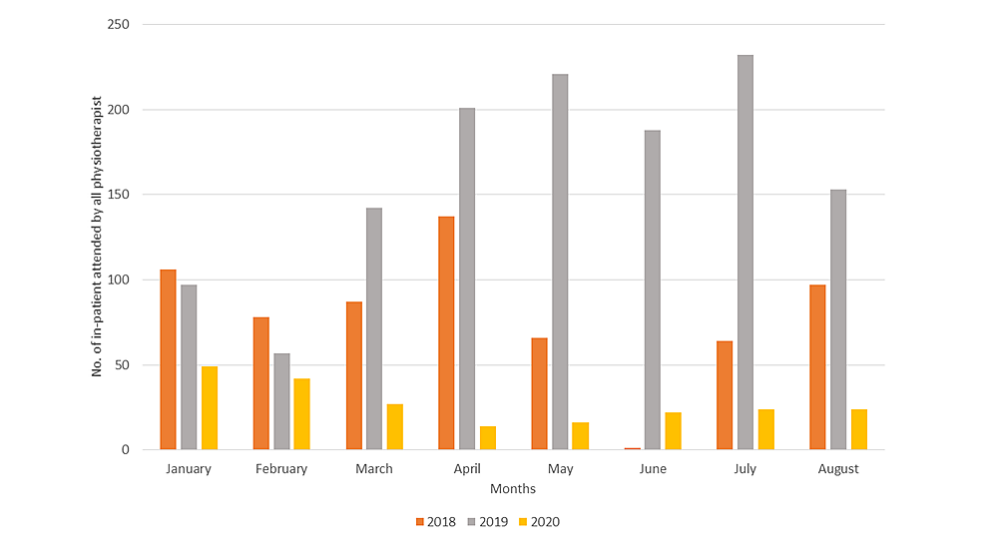
The number of patients treated by physiotherapy during selected periods in 2020 compared with the same periods in 2018 and 2019

The number of patients who received physiotherapy (Figure 3) in 2017 (median: 82.5, IQR: 65.5-92) also differed significantly from the number who received physiotherapy in 2020 (median: 24, IQR: 20.5-25.5, p=0.003). Similar results were found when 2020 was compared to 2019 (median: 170.5, IQR: 130.7-211, p=0.000).

No significant difference was found for the speech clinic data between the study period (median: 14.7, IQR: 72-57.5) and the same period previously (median: 30.25, IQR: 38.5-8.25).

## Discussion

This study aimed to investigate the impact of the COVID-19 pandemic on the provision of essential services involving multiple rehabilitation disciplines including speech therapy, occupational therapy, and physiotherapy.

The findings of this study showed a marked decline in the number of patient visits and sessions provided at the outpatient rehabilitation clinics at KFSHD due to the sudden ending of treatment and changed working circumstances during the lockdown from January to August 2020. As a result of the abrupt closing down of all non-critical and non-urgent medical services in hospitals treating COVID-19 patients, the rehabilitation services in multiple disciplines were significantly affected. Outpatient visits attended by physicians were at their lowest in May 2020 and the same was the case with inpatient services. Similarly, physiotherapists and occupational therapists attended the least number of in-hospital patients visits during the month of April 2020. A 77% drop in the number of patients treated in the physiotherapy clinics was observed from January to August in 2020 when directly compared to the same period in 2019 (pre-COVID-19 period).

With regard to the lockdown and reorganization of medical services that altered the accessibility to outpatient rehabilitation services, many reports from across the world have described the cessation or significant decline in outpatient services [15,16,17,18]. Our findings are in line with the results of the above-mentioned reports. For instance, Jesenšek Papež et al. (2021) have reported a 44% decline in the total number of patients and a 71% decline in the number of treatment sessions during the COVID-19 period 2020 compared to the pre-COVID-19 period from March-August in 2019 [18]. Likewise, Yağcı et al. (2020) have reported a decline of 84% and 92% in the number of weekly outpatient rehabilitation service visits and the number of patients seen at the time of the lockdown, respectively [19]. Altogether, these reports demonstrate the impact of the COVID-19 outbreak on rehabilitation services, which resulted in sudden cessation or substantial decline in outpatient rehabilitation service visits.

It is well documented that COVID-19 increases rehabilitation needs not only for infected patients with novel coronavirus but also for those who suffer from long-term sequelae of their diseases or disabilities [20]. The COVID-19 pandemic has resulted in a severe disruption of most rehabilitation services, which in turn has negatively affected all patients with disabilities or chronic conditions [12]. Yet, the entire range of problems arising after COVID-19 and its consequences are not known, given the fact that COVID-19 affects multiple body systems including respiratory, cardiovascular, neurological, musculoskeletal, gastrointestinal, and renal systems [21,22,23]. This means that post COVID-19, patients are at risk of developing a wide range of persisting impairments or dysfunctions across body systems, which may result in multiple disabling conditions physically and psychologically [24,25]. Thus, rehabilitation services should be continued throughout the COVID-19 pandemic or any other similar outbreak periods [26]; in addition, it should be integrated with COVID-19 medical management at different phases: acute, subacute, and long-term [22,25].

## Conclusions

We have clearly described the negative consequences of the COVID-19 pandemic on outpatient rehabilitation services in our study. Due to the pandemic, there was a sharp decline not only in the number of patients but also in the outpatient rehabilitation services such as physical and occupational therapy sessions. One of the limitations of our study was not taking into account the profiles of the patients, which would provide a better assessment. In addition, we took into account the number of patients and the number of outpatient rehabilitation services only, while other important parameters were not considered in this study. Further studies are warranted to analyze the impact of the COVID-19 pandemic on the functional status of patients as well as the ways in which delayed or obstructed rehabilitation services affect the patients' prognosis. Studies on the importance of timely rehabilitation are required to assess the consequences of such delays in the time of any pandemic. The major concern is that COVID-19 or any other similar pandemic in the future would obstruct rehabilitation patients’ healthcare services due to many factors. With a steeper rise in the number of patients with disabling conditions, the rehabilitation facilities may in the future confront the take-back effect, and the question is how we can be better prepared for such pandemics in the future and deliver appropriate and timely rehabilitation programs with a patient-oriented, tailored, and individualized type of rehabilitation design. Further large-scale and multiple-center studies are needed to understand the role of other factors including lifestyle and the scope of online rehab programs (telerehabilitation). Future emphasis might be placed on organizing virtual clinics with video facilities to reduce outpatient visits.
